# Case of Complete Occlusion of the Os Uteri

**Published:** 1854-09

**Authors:** B. N. Bond


ART. III.—*Case of Complete Occlusion of the Os Uteri.* By B. N. BOND, M.D.

Feb. 1, 1853.—I was called to Mrs. H. at 8. P M., in labor with her first child. She was an Irish woman, stout and large, aged 24 years; had strong pains since 4 P.M. The pains were urgent and powerful, forcing the head, covered by the uterus, low down into the vagina No os uteri could be felt; but about the centre of the tumor, and protruding from the vagina, could be felt a firm, hard point, with several ridges diverging from it. On inquiry I found that, in the August preceding she had a severe attack of remittent fever, followed by a *purulent*, *offensive* discharge from the vagina for several weeks after convalescence I at once concluded this to be a well-marked case of total occlusion, and requested consultation, which was sent for. I gave her an opiate, which put a stop to the pains till 4, A.M., when they returned with increased severity. Believing that, if left alone, rupture would take place before Dr. Jones could arrive, I at once proceeded to make an incision transversely across the hard point, about one and a-half or two inches in length. This dilated, and in ten minutes she was safely delivered of a fine healthy daughter. Recovery was slow, on acconnt of an offensive discharge from the vagina; but she finally recovered perfectly, and is now, July 30th, again pregnant, with every prospect of a happy termination. Believing that but few cases of *total* occlusion are met in practice, I have given a detail of all the facts of this, to me, new and interesting case.

